# First experiments with a water-jet plasma X-ray source driven by the novel high-power–high-repetition rate L1 Allegra laser at ELI Beamlines

**DOI:** 10.1107/S1600577521008729

**Published:** 2021-11-01

**Authors:** Anna Zymaková, Martin Albrecht, Roman Antipenkov, Alexandr Špaček, Stefan Karatodorov, Ondřej Hort, Jakob Andreasson, Jens Uhlig

**Affiliations:** a Structural Dynamics, ELI Beamlines, Za Radnici 835, Dolni Brezany 25241, Czech Republic; b X-ray sources, ELI Beamlines, Za Radnici 835, Dolni Brezany 25241, Czech Republic; c L1 Allegra Laser, ELI Beamlines, Za Radnici 835, Dolni Brezany 25241, Czech Republic; d Division of Chemical Physics, Lund University, Box 117, Lund 22100, Sweden

**Keywords:** X-ray generation, plasma X-ray sources, OPCPA lasers, compact X-ray sources

## Abstract

ELI Beamlines is a rapidly progressing pillar of the pan-European Extreme Light Infrastructure (ELI) project focusing on the development and deployment of science driven by high-power lasers for user operations. This work reports the results of a commissioning run of a water-jet plasma X-ray source driven by the L1 Allegra laser, outlining the current capabilities and future potential of the system. The L1 Allegra is one of the lasers developed in-house at ELI Beamlines, designed to be able to reach a pulse energy of 100 mJ at a 1 kHz repetition rate with excellent beam properties. The water-jet plasma X-ray source driven by this laser opens opportunities for new pump–probe experiments with sub-picosecond temporal resolution and inherent synchronization between pump and probe pulses.

## Abstract 2

An upgraded version of the water-jet plasma X-ray source suitable for handling the 100 W power of the driving 1 kHz optical parametric chirped-pulse amplification (OPCPA) L1 Allegra laser system has been installed and commissioned at ELI Beamlines. Hard X-ray generation with the L1 Allegra laser is demonstrated for the first time.

## 1. Introduction

The quest to study light-mediated processes has driven the development of facilities capable of producing ultrashort pulses of X-ray radiation (Ponseca *et al.*, 2017 ▸; Kranz & Wächtler, 2021 ▸; Chergui & Collet, 2017 ▸; Milne *et al.*, 2014 ▸). Laser-driven sources can reliably produce such radiation in a wide range of energies and combine the benefits of a compact setup with a high level of integratibility in a multi-purpose laboratory at affordable cost (in comparison with other large-scale facilities). For ultrafast pump–probe experiments, the all-optical approach of beam generation offers excellent synchronization between two or more beams. Such a facility has the potential for *e.g.* advanced shaped pump pulses (Assion *et al.*, 1998 ▸; Brüggemann *et al.*, 2006 ▸) and the intriguing capability for probes in different wavelength ranges, *e.g.* visible, terahertz and X-ray, using the same pump. The source described here is installed within a modular X-ray spectroscopy end-station, potentially motivating the use of multiple complementary methods for a comprehensive study [see De Roche *et al.* (2003 ▸), Naumova *et al.* (2018 ▸), Dicke *et al.* (2018 ▸), Kunnus *et al.* (2020 ▸) and Kjaer *et al.* (2019 ▸) for examples].

Laser-driven plasma X-ray sources (PXS) (Mallozzi *et al.*, 1974 ▸; Turcu & Dance, 1999 ▸; Benesch *et al.*, 2004 ▸) are based on focusing a laser with ultrashort (sub-100 fs) pulse durations and a peak intensity of 10^15^–10^17^ W cm^−2^ onto a renewable target (Fullagar, Harbst *et al.*, 2007 ▸; Korn *et al.*, 2002 ▸; Zamponi *et al.*, 2009 ▸; Uhlig *et al.*, 2013 ▸; Weisshaupt *et al.*, 2014 ▸; Afshari *et al.*, 2020 ▸). This results in ionization of surface atoms and plasma generation at the steep density gradient at the interface (Fullagar, Harbst *et al.*, 2007 ▸; Chen *et al.*, 2001 ▸; Brunel, 1987 ▸; Korn *et al.*, 2002 ▸; Uhlig *et al.*, 2011 ▸; Gibbon, 2005 ▸; Mulser & Bauer, 2010 ▸; Kruer, 2003 ▸; Uhlig *et al.*, 2013 ▸). Electrons in the plasma are subsequently accelerated by the electric field of the driving laser and finally interact with the target atoms, producing characteristic line emission (intense spectral peaks) and isotropic Bremsstrahlung radiation (continuous broadband background spectrum) (Brunel, 1987 ▸). The duration of the generated Bremsstrahlung X-ray pulse in the hard X-ray range (>4 keV) is mainly influenced by the duration of the laser heating (pulse duration of the driving laser) and the interaction path length (∼4–10 µm), and is typically >400 fs (Afshari *et al.*, 2020 ▸; Fullagar, Harbst *et al.*, 2007 ▸; Bargheer *et al.*, 2006 ▸; Pfeifer *et al.*, 2006 ▸). The complex processes in the interaction region and the wide range of applications led the research towards the development of many different target types and pulse conditions over the past two decades (Gibbon, 2005 ▸; Uhlig, 2011 ▸; Martynow *et al.*, 2019 ▸; Eliezer & Kunioki, 2019 ▸; Brauckmann, 2017 ▸; Macchi, 2013 ▸; Miaja-Avila *et al.*, 2016 ▸; O’Neil *et al.*, 2017 ▸; Koç *et al.*, 2021 ▸; Gibbon, 2005 ▸; Mulser & Bauer, 2010 ▸; Kruer, 2003 ▸).

The conditions and operational demands that make this facility unique and challenging are a high laser pulse energy (up to 100 mJ) and a high repetition rate (1 kHz), representing 100 W average power, combined with the demands of stability and flexibility required for a user facility that can be operated continuously over long periods of time.

At the end of 2019, a water-jet PXS was delivered and installed on the X-ray spectroscopy end-station at ELI Beamlines as a complement to the metal target X-ray sources currently being under development. The choice of a low-*Z* target combines the absence of characteristic lines (wide usable spectral range) with simplicity of experimental use, including nontoxicity of the target at room temperature, stability of operation, availability, and ease of debris handling (Martynenko *et al.*, 2021 ▸; Miaja-Avila *et al.*, 2015 ▸; Uhlig *et al.*, 2015 ▸; Uhlig, 2011 ▸). The compact experimental chamber with total dimensions 100 mm × 180 mm × 162 mm (W × L × H) makes the water-jet PXS an attractive candidate for the tightly packed multi-purpose X-ray spectroscopy end-station. Here we describe the commissioning run of this water-jet PXS driven by the 1 kHz L1 Allegra laser developed in-house, and assess its current feasibility for time-resolved experiments. The L1 Allegra laser utilizes the optical parametric chirped-pulse amplification (OPCPA) technique that combines high power with exceptional temporal contrast with the ability to shape the temporal structure (Batysta *et al.*, 2016 ▸) and optimize the dispersion of the laser beam according to the needs of a particular experiment.

The initial experimental run reported here was conducted in September 2020 and represents the first demonstration of hard X-ray generation with the L1 Allegra laser. The run was performed at a reduced laser power of 10–12 mJ per pulse under modal and chirp conditions that were non-optimal for the source. Neither position tracking nor alignment pinholes were available/used, although these measures are/will be implemented for future user operation.

PXS sources have shown a strong dependence on the electron temperature and integral flux of the driving power and on the precise interaction conditions (Miaja-Avila *et al.*, 2015 ▸; Fullagar, Harbst *et al.*, 2007 ▸). Based on these results, we will carefully estimate the perspective of the expected source X-ray flux and experimental conditions as the L1 Allegra laser approaches its design pulse energy of 100 mJ.

## 2. Experimental

The L1 Allegra laser system is based on a broadband OPCPA, pumped by picosecond Yb:YAG thin-disc lasers. After amplification, the broadband pulses are compressed to <15 fs (with the transorm limit around 12 fs) using a chirped mirror compressor. The system operates at a 1 kHz repetition rate with the central wavelength around 830 nm. At the time of writing, the L1 Allegra laser is in a ramp-up programme to reach 50 mJ for general user operations and has a maximum (design) value of 100 mJ. The laser is designed to be operated at multiple power levels, switching between a safe alignment mode and the high-power operation. For the present experiments, the system was used at a pulse energy level in the range of 10–12 mJ and intensity contrast of 10^−9^ on the picosecond scale (Antipenkov *et al.*, 2019 ▸). In the large ELI Beamlines facility, the laser is located in a different room on a different floor, with an in-vacuum beam path of ∼40 m from the last mirror of the L1 Allegra injector to the experimental station. The beam transport is done in high vacuum (10^−6^ mbar; 1 bar = 100 000 Pa) on four-inch optics.

**Figure 1:**
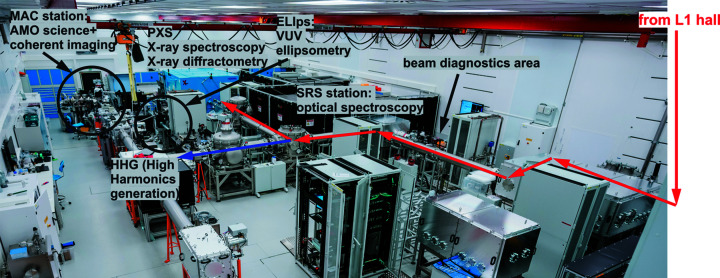
General layout of E1 experimental hall and L1 Allegra laser beam transport. Red arrows show Allegra beam transport to the water-jet PXS.

A general view of the beam transport and experimental areas in the E1 experimental hall is shown in Fig. 1 ▸. The L1 Allegra laser is sent from the L1 hall, located above E1, and can be directed to one of two types of secondary sources – an HHG source for XUV radiation (Hort *et al.*, 2019 ▸, 2020 ▸) or plasma X-ray sources (Nejdl *et al.*, 2019 ▸). The HHG source serves two stations for XUV science: ‘MAC’ for atomic, molecular and optical science and coherent diffractive imaging (Klimesová *et al.*, 2021 ▸), and ‘ELIps’ for XUV materials science applications (Espinoza *et al.*, 2020 ▸). Complementary methods for ultrafast optical spectroscopy are also available (using support lasers): transient optical absorption (Naumova *et al.*, 2018 ▸), stimulated Raman scattering (Andrikopoulos *et al.*, 2020 ▸), time-resolved spectroscopic ellipsometry (Richter *et al.*, 2021 ▸) and IR (1D and 2D) spectroscopy.

For the water-jet PXS, inside a small safety hutch designated for X-ray experiments, the laser beam is coupled out of the vacuum transport through a CF100 window. The power and polarization of the driving laser are adjusted by a half-wave plate and two Brewster windows in an Altechna laser attenuator, ensuring horizontally polarized light at the inter­action region, and finally focused by a three-inch 90° off-axis parabolic (OAP) mirror with 15 cm focal length. Most of the group velocity dispersion introduced through all optical elements in the beam path is then compensated through optimization of the supercontinuum created by the focused beam in air. The effects of pulse compression and other laser parameters have been discussed previously (Fullagar *et al.*, 2008 ▸; Uhlig, 2011 ▸).

**Figure 2:**
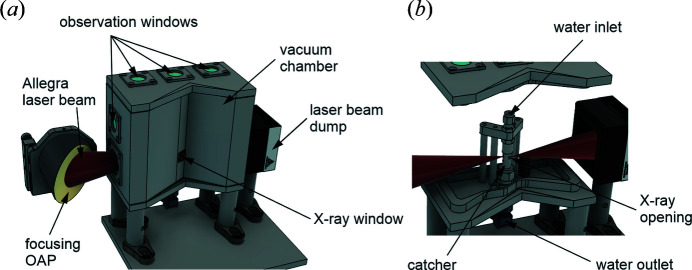
Schematic diagrams of water-jet PXS optimized for the high-performance L1 Allegra laser. Water is continuously recycled in a closed-loop pumping system with inlet and outlet reservoirs (not shown in the scheme). The water jet is shaped by a nozzle in the water inlet area and driven by a pressure difference.

The water-jet PXS chamber is based on existing design iterations (Fullagar, Harbst *et al.*, 2007 ▸; Uhlig *et al.*, 2013 ▸; Uhlig, 2011 ▸) and was adapted for use with the high-power beam from the L1 Allegra laser (Zymaková *et al.*, 2020 ▸). The combination of the high power and high repetition rate of the laser can result in damage to the optical components, strong cooling of the chamber due to increased evaporation can lead to the formation of ice, and the high-repetition rapid expansion of gas/water created by the focused laser beam increases the debris load. The upgraded design (see Fig. 2 ▸) includes increased optical apertures for damage mitigation at the anti-reflection coated windows.

An external beam dump located on the opposite side of the laser entrance window terminates the high-pulse-energy beam of the L1 Allegra laser and is thermally coupled to the water jet catcher/outlet. This transfers a part of the laser energy to the chamber, effectively preventing ice formation, reducing water condensation on the windows, and supporting dissipation of 100 W average power ultimately expected from the laser. A flexible and improved debris handling system confines the mist with dedicated flow channels, and a tight design allows outcoupling of the X-ray radiation close to the source (<1.2 mm) without exposing the window material to potential reflections of the laser. The vacuum chamber contains strategically placed windows to ease alignment and allow inspection of the target jet and the laser-jet interaction zone. Two cameras with magnifying optics are installed for observation of the laser focus and interaction region from two perpendicular directions. Under usual operation conditions, the optical elements before the chamber consist of a modular beamsplitter and an OAP mirror that is initially optimized and then kept static. The X-ray source chamber including the water jet is remotely positioned relative to the laser focus, controlling the interaction region.

The X-ray source and all X-ray optical elements, like an X-ray polycapillary lens (Zymaková *et al.*, 2020 ▸), are mounted on a common base plate and move in conjunction with the chamber and the rigidly mounted water jet, maintaining their relative alignment. The sample section and the focusing lens of the pump beam are either mounted on the same baseplate or independently, depending on the experiment. The target is a cylindrical 100–300 µm thick water jet formed by a high-performance liquid chromatography (HLPC) nozzle at a typical speed of ∼6 m s^−1^ driven by a 1–2 bar pressure difference between a pressurized reservoir and the evacuated experimental chamber. The setup uses two identical reservoirs each lasting for 10–12 h of operation, the current facility-limited working time. The flow design allows for fast reservoir switching and near-continuous operation.

The interaction region is typically located on the jet 3 mm below the opening of the HLPC nozzle, ensuring a complete replacement of the interaction/source region surface for each pulse of the driving laser and a very smooth surface. The water is degassed in vacuum and pressurized with industry-quality helium. These measures prevent instability of the jet as it enters the target chamber (Otendal *et al.*, 2005 ▸), which is verified by direct inspection with a microscope and observation of the laser beam reflected from the jet surface.

The water-jet PXS vacuum chamber is mounted on two perpendicular motorized stages from Physik Instrumente (PI), with travel ranges of 52 mm along the laser-beam direction and 102 mm transverse to the laser beam, a minimum incremental motion of 0.5 µm and unidirectional repeatability of 0.5 µm. The OAP is optimized to generate the brightest spark at a low laser pulse energy in the focus in air after removing the chamber from the beam path. After initial placement of the chamber and coarse alignment through the observation windows, fine alignment utilizes the shape and position of the plasma plume created by the interaction of the laser with the water jet, as observed with web cameras.

The region of optimal X-ray production is defined by the rigid jet assembly and the optimally focused laser beam and has an extension of typically <5 µm perpendicular to the laser-propagation and jet-flow direction and <50 µm along the laser-propagation direction, imposing high demands on the spatial stability of the laser focus (Gustafsson, 2015 ▸; Fullagar *et al.*, 2008 ▸). During operation, the position of the PXS is optimized using the integrated flux measured with the X-ray CCD and a RadEye detector as feedback. The RadEye detector is a large-area (position-insensitive) Geiger–Mueller counter used as a pseudo-linear detector at counting rates far below the maximum of 1 kHz, temporarily replacing a more suitable linear detector for alignment purposes.

The data presented here were collected by an Andor Newton X-ray CCD camera with an e2v CCD30-11 front-illuminated deep-depletion chip in an indirect detection mode. In this camera, the CCD is Peltier-cooled to 233 down to 223 K and kept in a low-pressure protective gas close behind a beryllium window. In this configuration, the CCD acts as a large assembly of X-ray diodes. The challenge of this configuration is that each frame can only contain a limited number of absorption events and some X-ray-generated electron clouds are only partially registered due to the reduced active space. This can, under certain circumstances, lead to spectral redistribution and charge sharing among adjacent pixels (Lutz, 2006 ▸; Bautz *et al.*, 1999 ▸; Pavlov & Nousek, 1999 ▸; Janesick, 2001 ▸; Fullagar *et al.*, 2008 ▸; Uhlig, 2011 ▸).

We follow the previously published and verified procedure to calibrate, extract and correct the spectrum from the collected full-frame images (Fullagar *et al.*, 2008 ▸; Uhlig, 2011 ▸). During the experiment, the X-ray source was operated continuously at 1 kHz without a shutter between the source and detector. To reduce the influence of different voltage configurations in the readout and exposure mode employed by this type of CCD sensor, the exposure time was chosen as 2 s per frame. For the regular operation of such a characterization method, a camera shutter, a laser pulse shutter or a frame storage/transfer approach on a larger chip could be employed to allow for significantly shorter exposure times and subsequent faster spectral feedback.

Each absorption event results in a de-excitation charge cascade, creating a charge cloud of characteristic value for the photon energy that is then digitized to a number. In the current configuration and in the absence of partial registry, typically one analogue-to-digital (AD) conversion unit corresponds to ∼10 eV of the initial photon energy. The measured AD values are then corrected by the background and calibrated by known emission lines.

The highest energy resolution is achieved by suppressing events that are spectrally redistributed, which is often achieved by suppressing absorption events that extend beyond a single pixel (Fullagar *et al.*, 2008 ▸; Uhlig, 2011 ▸). The front-illuminated e2v sensor used in this experiment has an approximate depletion depth of 40 µm (determined by modelling the observed response function) which, in combination with the large pixel size, leads to a small fraction of redistributed events. The total intensity of the measured spectrum is then deduced by normalizing the observed event histogram to the expected quantum efficiency of the deep depletion layer at each energy. For this experiment, the difference between the number of events observed in a single pixel and the total number of events observed in any number of pixels is usually kept below 5%, or 20% if each exposed pixel is counted as a separate event, supporting this approach. The challenge of this approach is a significantly limited maximum number of absorption events (typically 1/200 pixel) recorded by the camera.

For the analysis, we have implemented the following procedure. For each pixel, an individual background level is subtracted using the statistics over 800 frames to account for spatial temperature differences due to cooling gradients over the chip area. Then a common background level for each frame is determined and subtracted to account for background variations over the progression of the frames due to temperature variations induced by the continuous exposure to X-ray radiation and readout. A threshold is applied to the data corresponding to five times the σ of the background model (assuming a Gaussian distribution). Histograms of the analogue-to-digital conversion (ADC) steps are then created for all pixels and for the absorption events contained within a single spatially isolated CCD pixel (Fullagar *et al.*, 2008 ▸; Uhlig, 2011 ▸). The ADC units in the histogram are then converted into energy units by aligning the (fitted) peak of the measured *K*_α_ emission of an iron calibration foil to 6405.2 eV. The observed intensity is then converted into a photon flux accounting for accumulation time, laser repetition rate and the solid angle of the detection setup. Finally, the number of detected photons is corrected by the detection probability for the corresponding setup. This probability includes the absorption probabilities of all materials in the beam path, from the source to the detector for the absorption measurement (with the exception of the sample) or from the sample to the detector for the emission measurement, and in both cases the quantum efficiency of the detector. These energy-dependent correction factors used database values from the *xraylib* (Schoonjans *et al.*, 2011 ▸) broadened by a Gaussian kernel to represent the CCD’s inferred X-ray energy resolution. The integrated flux of the X-ray source is sufficiently strong that spatial constraints in the X-ray hutch necessitate accumulation with a (copper) filter in the direct detection method [X-ray absorption spectroscopy (XAS)] that is then compensated as part of the data analysis. The effective removal of the Cu *K* absorption edge (without optimization of the Cu thickness parameter) increases the trust in our compensation procedure.

**Figure 3:**
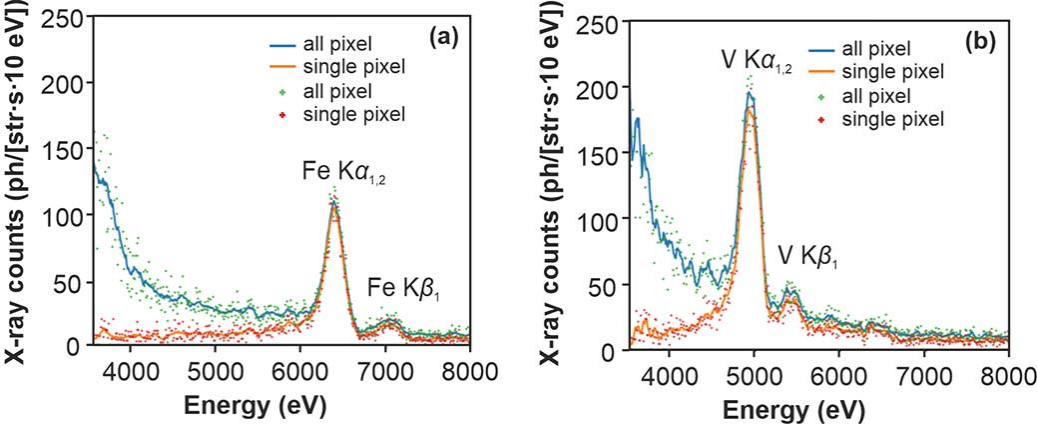
X-ray emission spectra of (*a*) iron and (*b*) vanadium acquired using EXAFS Materials calibration foils. (The thicknesses of the foils are 7.5 µm for Fe and 5 µm for V.)

These commissioning experiments were performed to characterize the performance and demonstrate the generic suitability of this laser-based X-ray spectroscopic setup for pump–probe experiments. The long path in air and the copper foil place a severe limitation on the usable spectral range shown in Fig. 3 ▸(*a*). Future experiments with a focus on analysis of the laser–plasma interaction should employ alternative methods to limit the X-ray flux on the detector (see above) and replace the air in the beam path with *e.g.* helium to extend the accessible spectral range.

### 2.1. Characterization of this source for X-ray emission spectroscopy

A metal foil from EXAFS Materials was placed close to the X-ray window as an initial target. The illuminated area was a round spot of 2 mm defined by an aperture at a total distance of ∼42 mm from the source. This distance was limited by the geometry of the target chamber (Fig. 2 ▸) (the closest position after the window/aperture would be ∼18 mm from the source). The induced X-ray fluorescence emission was collected on the CCD at a distance of 210 mm from the foil and at an angle of approximately 90° to the incident X-ray beam. This position is close to the typical placement of a bent von Hamos type analyser crystal using Si for Cu *K*_α_ X-ray emission spectroscopy (XES) observations (Szlachetko *et al.*, 2012 ▸), one of the analyser geometries planned for the setup. For each spectrum we collected 800 images in two sets, maintaining the general camera settings and alignment. The resulting XES spectra were extracted using the method described above and are shown in Fig. 3 ▸. The observed flux is binned into 10 eV bins to represent the approximate width that would be used for high-resolution XES measurements (typically 10–40 eV). The expected Fano width of ∼110 eV was convolved with the baseline width (from the background) and is approximately the resolution observed in the CCD data.

### 2.2. Characterization of the direct flux and X-ray absorption spectrum

**Figure 4:**
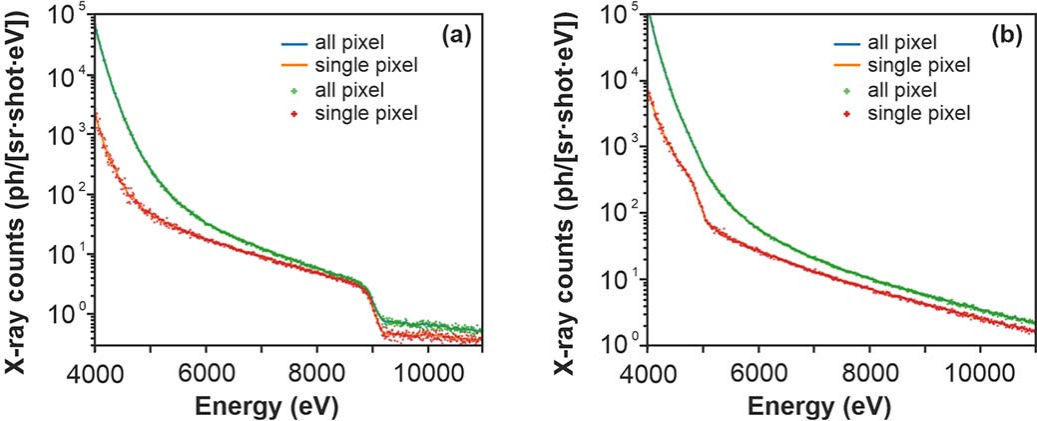
Direct CCD observed X-ray absorption spectra of (*a*) copper and (*b*) titanium acquired using EXAFS Materials calibration foils. The thicknesses of the foils are 7.5 µm for Cu and 6 µm for Ti. The shown flux is compensated for solid angle and all materials between source and detector except for the foils themselves.

The camera was placed at a distance of 915 mm in the line of sight of the X-ray window for direct X-ray detection, through air. The absorption sample was placed close to the source, to avoid the influence of fluorescence photons, and potential sources of secondary radiation were carefully removed from the beam path. The exposure time and camera distance were chosen to allow for collection of the radiation while maintaining a sufficiently low number of exposed pixels to use the camera as a direct detector. In each experiment, we collected 800 images in two sets, while the camera parameters and alignment were kept unchanged. The spectra in Fig. 4 ▸ clearly show the ability of the source to provide a sufficient number of photons to accumulate X-ray absorption spectra over a wide range of energies. The low-energy limit of these spectra is defined through the absorption of air that at 91 cm and 4 keV transmits only 0.01% of all X-rays.

### 2.3. Discussion of average and peak flux

The experiment in this paper describes the first use of the L1 Allegra laser to drive a water-jet PXS and, to the best of our knowledge, the first reported use of such a high-power 1 kHz OPCPA laser system to drive this type of X-ray source for the creation of broadband radiation. The emission and absorption spectra in Figs. 3 ▸ and 4 ▸ were accumulated over an effective time of 2 × 13 min, in the case of XAS limited mainly by the speed of the camera. If the spectra are also compensated for the sample foil, we can estimate the achieved flux to be 260 photons per shot sr^−1^ eV^−1^ at 6 keV (corresponding to 1.5 × 10^6^ ph sr^−1^ s^−1^ per 0.1%BW) during a subset of the frame from 100 s. This is not far from the expected value for the combination of laser power and source conditions used here if we use prior experiments as reference (Miaja-Avila *et al.*, 2015 ▸; Uhlig *et al.*, 2013 ▸). Visible drifts of the laser beam during the accumulation period significantly reduced the flux over the progression of frames (which was the reason for the changing background levels). Actively (manually) following these movements through small steps in the 2–5 µm range using the motorized stages, and the integral X-ray flux as a monitor, enabled us to maintain the observed peak flux over a longer period. While the inclusion of an active feedback system would be a viable option, it is hoped that the currently introduced active beam stabilization will remove this need.

**Figure 5:**
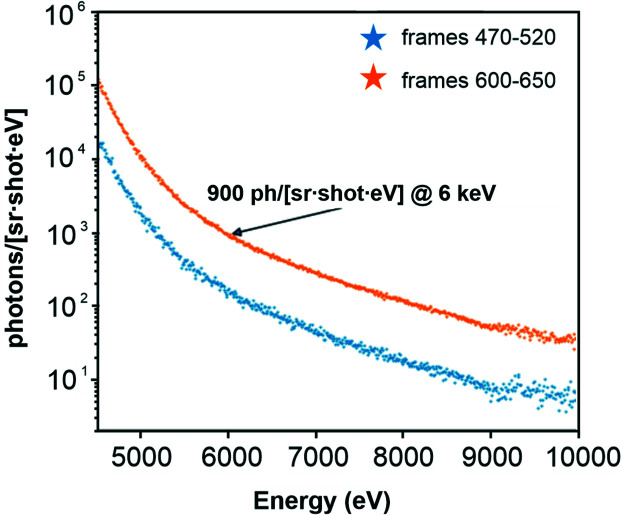
Two selected sets of data, each containing 50 frames, clearly demonstrating the achieved photon flux (orange) and the effect of driving laser instabilities (blue). A flux of 900 ph sr^−1^ shot^−1^ eV^−1^ at 6 keV (corresponding to 5.4 × 10^6^ ph sr^−1^ s^−1^ per 0.1%BW at 6 keV) was observed after source optimization.

Fig. 5 ▸ shows the spectrum for the maximum flux reached shortly after one such adjustment. The integrated flux is 3.5 times higher than the average output used for accumulation of the spectra in Figs. 3 ▸ and 4 ▸. This factor is an estimation gained from the integral flux observed on the CCD camera. Previous studies (Fullagar, Uhlig *et al.*, 2007 ▸; Fullagar *et al.*, 2008 ▸; Miaja-Avila *et al.*, 2015 ▸) indicate that the conditions at the interaction point influence the electron temperature, and with it not only the total emitted flux but also the emitted spectrum. Under the conditions in this experiment, we preferentially record photons with higher photon energy (>8 keV). The electron temperature, and with it the fraction of emitted high-energy photons, is reduced if the focal conditions are worse, which is why we feel comfortable using this factor as an estimate for the flux at 6 keV. Factoring in this effect the achieved peak emission is >900 photons per shot sr^−1^ eV^−1^ at 6 keV (corresponding to 5.4 × 10^6^ ph sr^−1^ s^−1^ per 0.1%BW at 6 keV).

For the experiment presented here, 10 to 12 mJ were used to drive the water-jet PXS. The L1 Allegra laser is under rapid development and has recently demonstrated an ability to reach a pulse energy of >50 mJ (at the laser output). It is designed to reach 100 mJ (Batysta *et al.*, 2016 ▸) with discussions currently underway for further increase. It may be anticipated that operating the water-jet PXS at increased pulse energy will also result in increased X-ray flux. Currently, an upgrade of the water-jet PXS vacuum system is being implemented that will improve the vacuum conditions, which have shown to be critical for reducing self-phase modulation and thus the achievable maximum laser fluence (Thoss, 2003 ▸; Mourou *et al.*, 2006 ▸). The effects of an upgraded vacuum system and an improved laser focus through the reduction of the current astigmatism are difficult to predict. However, the implementation of active and passive beam stabilization and, if still necessary, the use of an active feedback system for the target position will stabilize the already measured 3.5-fold flux increase. If we estimate the increased X-ray flux through the ten-fold higher laser power and the other updates with a very conservative factor of 4, after implementation of multi-aspectual upgrades we can expect at least a 14-fold increase in flux to >3600 photons per shot sr^−1^ eV^−1^ at 6 keV (corresponding to 2.2 × 10^7^ ph sr^−1^ s^−1^ per 0.1%BW) together with a raised electron temperature.

Very recent work from the Elsaesser group used an OPCPA as a driving laser with 5 µm wavelength and created an X-ray source with copper tape for generation of Cu *K*α emission. They achieved in comparable units 9.5 × 10^9^ ph sr^−1^ s^−1^ using the superior generation of hot electrons at a longer laser wavelength (Koç *et al.*, 2021 ▸). Based on our numbers and assuming an active bandwidth of >1 keV for generation of the core hole in the hard X-ray range (*e.g.* iron), we can expect an X-ray emission flux on the same scale from our water-jet target.

Based on the observed numbers and reasonable improvements, we can estimate the conditions for experiments performed with this source. For a cylindrically bent crystal in von Hamos geometry with a 25 cm focal distance under normal incidence, we can assume for the 1 eV energetic acceptance a 12 µm wide acceptance stripe in the dispersive direction, which corresponds to a solid angle of 20 µsr. With a crystal size of 10 cm in the non-dispersion direction, we can expect approximately 17 ph s^−1^ eV^−1^ at 6 keV on the detector, not including losses due to air absorption and reflectivity efficiency. As the source is broadband and the full spectral window is collected simultaneously, the full spectrum is accumulated at a comparable rate. For a differential spectrum 10^5^ photons per energy bin can be sufficient, assuming 1% fluctuation of the pump laser as the main source of noise. This corresponds to an expected accumulation time of approximately >2 h for a full transient spectrum in the range of the iron *K* edge, which is reduced to <10 min after the planned improvements. Similarly, at the observed maximum flux for iron foil emission, a rough estimate yields 1 ph s^−1^ on the detector over the full spectrum when collecting nearly background-free emission spectra (*K*_α_). A usable spectrum can be achieved with 6000 photons (2 h accumulation time) reduced to <10 min at the conservatively improved conditions. The often targeted transition metal *K*_β_ lines have a 20-fold lower emission probability and will only be accessible after the improvements.

Further reductions in the accumulation time in X-ray spectroscopy experiments could also be pursued based on adaptations of the spectrometer design. The beam path of XAS measurements is limited by the opening angle of the experiment and thus cannot be significantly increased. In contrast, the recently deployed multiple crystal detection schemes (Alonso-Mori *et al.*, 2012 ▸; Kalinko *et al.*, 2020 ▸) would allow a six-fold decrease in the accumulation time. Other developments, such as the use of energy-dispersive cryogenic detectors, could reduce the accumulation times even more (Doriese *et al.*, 2017 ▸; O’Neil *et al.*, 2017 ▸; Uhlig *et al.*, 2015 ▸; Miaja-Avila *et al.*, 2016 ▸). Low-temperature detectors, such as microcalorimeters, analyse the energy of individual photons without the use of optical elements and found their first use in the field of ultra-fast spectroscopy together with an earlier generation of the water-jet PXS (Fullagar *et al.*, 2008 ▸; Uhlig *et al.*, 2013 ▸; Uhlig, 2011 ▸).

## 3. Summary and outlook

In summary, we have shown that at this early stage the L1 Allegra laser combined with a water-jet PXS can deliver sufficient X-ray flux to perform proof-of-principle experiments in X-ray emission and absorption spectroscopies, and we have outlined a pathway that will transform water-jet PXS within the ELI Beamlines X-ray spectroscopy end-station to become a valuable user facility for time-resolved pump–probe studies.
